# Penile Hair Tourniquet Syndrome (PHTS): A Case Report of a Two-Year-Old Boy

**DOI:** 10.1155/2022/8030934

**Published:** 2022-08-09

**Authors:** Alhareth Baarimah, Latif Dar, Rayan Dashnan, Saeed Alshahrani, Mohammed Beaiti, Khaled ALDhabaan

**Affiliations:** ^1^Department of Urology, International Medical Center (IMC), Jeddah, Saudi Arabia; ^2^Department of Pediatric Urology, Abha Maternity and Child Hospital (AMCH), Abha, Saudi Arabia; ^3^Department of Pediatric Urology, Khamis Mushayt Maternity and Children Hospital, Khamis Mushayt, Saudi Arabia

## Abstract

Penile hair tourniquet syndrome (PHTS) is an unusual phenomenon. A physician should have a high index of suspicion when a circumcised child presents with glans swelling and inflammation. It must be considered a surgical emergency, as early diagnosis and treatment can prevent complications (e.g., urethra-cutaneous fistula, complete urethral transection, penile gangrene, and penile amputation). We report a case of two-year-old boy to highlight the importance of early diagnosis and prompt treatment.

## 1. Introduction

Hair tourniquet syndrome (HTS) is a condition seen mostly in circumcised children. It is characterized by a constriction caused by hair around an appendage, clitoris, or penis, and its clinical picture is akin to a compartment syndrome. When it involves the glans penis, it is referred to as penile hair tourniquet syndrome (PHTS) or hair coil penile strangulation [1]. The hair gets wrapped around the coronal sulcus of a circumcised child, leading to a spectrum of clinical situations ranging from mild swelling and redness of the glans to more serious complications such as urethro-cutaneous fistulas, gangrene, and penile amputation [2]. We are reporting this case because a delay in diagnosis—due to a lack of awareness among general practitioners, or uncareful examination—can lead to devastating complications [3].

## 2. Case Presentation

A two-year-old boy was admitted to the ER with a painful swelling of the penis, which his mother had first recognized three days prior. A pediatrician had prescribed a local antibiotic cream and analgesic, but the condition had not improved. The boy had begun to have difficulty passing urine.

**Figure 1:**
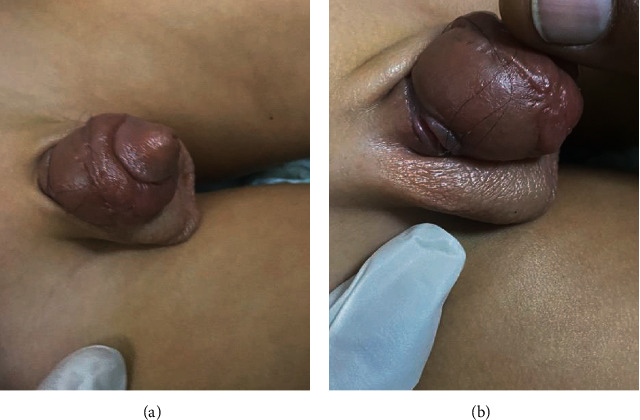
(a) Appearance of penile hair tourniquet syndrome at time of diagnosis. (b) Causative factor: hair strand of mother.

Upon arrival at the ER, the patient's general physical examination was normal. Upon local examination, the penis was found swollen, edematous, and tender to the touch. The glans penis was somewhat dusky. A constriction band was noticed at the base of the penis (Figure 1). The boy was reexamined using a loupe, and a hair coil was found embedded within the constriction mark. A local anesthetic spray was applied. The hair was held with a blunt-tipped probe and cut using microscissors. After removing the constricting hair coil, the area was carefully examined. The skin and subcutaneous tissue appeared eroded—and more so on the ventral aspect of the penis. An 8F Foley catheter was passed, and the urethra was found intact. The catheter was kept in place, and the patient was administered oral antibiotics and anti-inflammatory medication.

**Figure 2:**
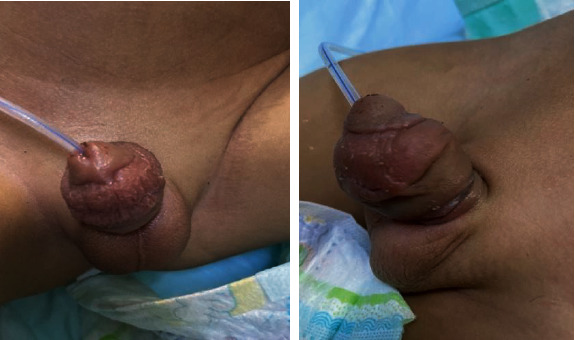
Postoperative day three with the catheter in place.

**Figure 3:**
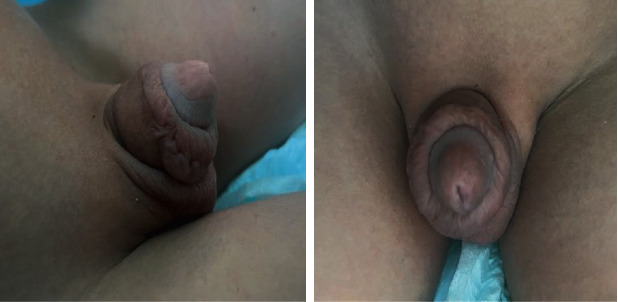
At one month, the wound had completely healed without any complications.

The patient was discharged home on postoperative day three with the catheter still in place (Figure 2). After removal of the catheter five days later, the patient passed urine freely without any difficulty. At one month, the wound had completely healed without any complications (Figure 3).

## 3. Discussion

PHTS is a well-established phenomenon that affects children. It is characterized by strangulation of the glans penis by a constricting hair coil. Although frequently described in relation to the penis in circumcised boys, the penis is not the only affected organ: HTS can involve any appendage with an end artery, including fingers, toes, and the clitoris. Cases affecting the vulva, labia minora, and ear lobules have also been reported [4, 5].

If it goes unrecognized, the condition can lead to devastating complications [2]. It is easily preventable, and when recognized early, it can be treated simply by removal of the coil under local anesthesia [3]. The problem is lack of awareness: PHTS usually goes unnoticed because most primary care providers, such as pediatricians, emergency pediatric physicians, and pediatric surgeons, are not fully aware of the condition [1, 4].

The first case was reported as early as 1832, but, surprisingly, the condition still has not received the recognition it deserves. It is barely mentioned in textbooks, although numerous case reports have been published in the literature [3–5].

PHTS is almost exclusively seen in children—and most frequently among those between one and eight years of age. Infants are especially vulnerable [4]. Cases have been reported in toddlers, school-age children, and even later in life [1]. Circumcision is an important predisposing factor [1–3]. The usual culprit is the mother's hair, which can get into a baby's diaper and become wrapped around the penis [3]. This is usually accidental, but some cultural beliefs may also play a part, as hair may be intentionally placed around the penis in the belief that it will improve sexual performance in adult life, treat enuresis, or get rid of evil spirits [2–5].

Once the coiled hair dries, it constricts and strangulates the distal penis. Initially, lymphatic and venous backflow is affected, leading to edema and swelling of the distal penis. This progresses and eventually affects arterial flow, resulting in ischemia, necrosis, gangrene, and amputation of the affected part of the penis [1, 3]. In 1980, Bashir and El-Barbery described the morphological characteristics of PHTS and classified its degrees of injury into four grades (Table 1) [4]. In our case, the injury falls into grade 0.

The diagnosis of PTHS is not straightforward, especially in the presence of swelling and edema. The hair becomes buried in the skin and is difficult to detect with the naked eye. A high degree of suspicion and awareness is therefore essential [3, 4]. Magnifying glasses or loupes are useful aids for detecting the constricting hair coil [3].

Once the diagnosis is made, treatment involves removing the constricting band of hair by cutting or unwinding it [4]. While using a magnifying glass or a loupe, the hair is held with a blunt-tipped probe and cut using microscissors [3]. The procedure can be performed in the ER or the outpatient clinic using a local anesthetic spray [3]. Depilatory hair-removal creams have also been used. If removal fails, a surgical exploration under anesthesia becomes necessary [3].

## 4. Conclusion

PHTS is an unusual phenomenon. Early recognition and prompt treatment are necessary to prevent devastating, serious complications. This condition frequently goes unrecognized due to a lack of awareness among primary caregivers. A high degree of suspicion and raised awareness will go a long way to ensuring early diagnosis, prompt treatment, and prevention of PHTS complications.

## Figures and Tables

**Table 1:** Classification of the degree of penile injury.

Grade	Description of the injury
Grade 0	Constriction of skin, without urethral injury
Grade I	Partial division of the corpus spongiosum and occurrence of a urethrocutaneous fistula
Grade II	Complete division of the corpus spongiosum and constriction of the corpus cavernosum
Grade III	Gangrene, necrosis, and complete amputation of the glans penis

## Data Availability

Data is available within the manuscript.
